# Mobile health contingency management for smoking cessation among veterans experiencing homelessness: A comparative effectiveness trial

**DOI:** 10.1016/j.pmedr.2023.102311

**Published:** 2023-07-03

**Authors:** Sarah M. Wilson, Dan V. Blalock, Jonathan R. Young, Sarah C. Griffin, Jeffrey S. Hertzberg, Patrick S. Calhoun, Jean C. Beckham

**Affiliations:** a VA Center of Innovation to Accelerate Discovery and Practice Transformation (ADAPT COIN), Durham Veterans Affairs Health Care System, Durham, NC, United States; b Department of Psychiatry and Behavioral Sciences, Duke University School of Medicine, Durham, NC, United States; c Mid-Atlantic Mental Illness Research, Education, and Clinical Center (MIRECC), Durham Veterans Affairs Health Care System, Durham, NC, United States; d Duke Clinical Research Institute (DCRI), Duke University School of Medicine, Durham, NC, United States

**Keywords:** Smoking cessation, Homelessness, Insecure housing, Veterans, Contingency management, Financial incentives, Mobile health

## Abstract

Tobacco cessation is reduced in U.S. military veterans experiencing homelessness. Mobile contingency management (mCM) is a promising treatment for tobacco use among populations experiencing homelessness, but past CM studies have largely been small, have relied on in-person follow-up, and/or lacked long-term biochemically verified abstinence measures. Veterans who smoked and were experiencing homelessness (N = 127) were randomly assigned to mCM treatment (4 weeks of mCM, 5 weeks of telehealth counseling, and the option of 12 weeks of pharmacotherapy) or VA standard care (3 biweekly group sessions and clinically appropriate pharmacotherapy), and all participants were randomly assigned to a $100 longer-term financial incentive for abstinence at 3-month follow-up. Participants were followed at 3-, 6-, and 12-months post-randomization, with the a priori main outcome designated as biochemically verified prolonged abstinence (with lapses) at 6-month follow-up. At 6-months, participants in the mCM group were significantly more likely to meet criteria for prolonged abstinence (*OR* = 3.1). Across time points, veterans in the mCM group had twice the odds of prolonged abstinence as those in the standard care group. However, by the 12-month follow-up, there was no statistically significant group difference in abstinence. Cost-effectiveness analysis indicated a modest increase in cost ($1,133) associated with an increase of one quality-adjusted life year saved for the intervention compared to standard care. mCM is a cost-effective approach to smoking cessation among veterans experiencing homelessness. Considering waning potency of this and other tobacco cessation interventions at 12-month follow-up, it is crucial to implement strategies to sustain abstinence for individuals experiencing homelessness.

## Highlights

• This was a study of smoking cessation for Veterans experiencing homelessness.
• We tested the effectiveness of mobile contingency management compared to standard care.
• At six-month follow-up, Veterans in the intervention group were more likely to be abstinent.
• The intervention only cost $1,133 more to save one quality-adjusted life year compared to control.
• Treatment effects waned over time.

## 1 Introduction

Cigarette smoking among U.S. military veterans experiencing homelessness is a pressing health equity issue. Although veterans experiencing homelessness have high risk for tobacco use, cessation interventions in this population are not widely studied and have shown limited efficacy (Tsai et al., 2011, Tsai and Rosenheck, 2012, Vijayaraghavan et al., 2020). Contingency management (CM) may be a cost-effective way to maximize smoking cessation efficacy among veterans experiencing homelessness. CM is a behavioral therapy that provides reinforcers (e.g., money) to individuals contingent upon evidence of a certain behavior (e.g., abstinence). Both CM and longer-term financial incentives are effective interventions for tobacco abstinence (Notley et al., 2019, Secades-Villa et al., 2020, Wilson et al., 2018).

Given its efficacy in achieving initial smoking abstinence (Notley et al., 2019, Secades-Villa et al., 2020), CM is theorized to be helpful for individuals experiencing homelessness, especially when combined with other efficacious treatments (Businelle et al., 2014, Carpenter et al., 2015). The existing evidence for CM for tobacco cessation among smokers experiencing homelessness is limited by two main factors: 1) small samples and 2) lack of long-term follow up past 6 months (Baggett et al., 2018a, Baggett et al., 2018b, Rash et al., 2018). In previous studies, biochemical verification of abstinence required frequent in-person visits, which are burdensome for facilities and patients (Baggett et al., 2018a, Baggett et al., 2018b, Rash et al., 2018). However, technology has allowed for the development of mobile CM (mCM), which enables remote abstinence verification (Hertzberg et al., 2013). A recent *meta*-analysis indicated efficacy of mCM for substance use disorders (SUD; Getty et al., 2019). Furthermore, findings from a small, non-randomized pilot trial of mCM for veteran smokers experiencing homelessness indicated that 30% of veterans who received the treatment achieved seven-day point prevalence abstinence at 6 month follow-up (Carpenter et al., 2015).

Additionally, the effectiveness of longer-term financial incentives for cessation (i.e., larger sums of money delivered at a more remote time point upon verification of abstinence) has been demonstrated in the general population (Notley et al., 2019) but is understudied within those who smoke and experience homelessness. Moreover, there are few studies testing the combined effectiveness of short- and longer-term incentives combined (Carpenter et al., 2015).

The current study assessed the effectiveness of an intervention designed for homeless veterans that consisted of mCM, telephone-delivered cognitive behavioral therapy (CBT), tobacco cessation pharmacotherapy, and long-term incentives for abstinence. The primary study aim was to evaluate the effectiveness of the intervention on biochemically verified prolonged smoking abstinence at 3-, 6-, and 12-months post-randomization. We hypothesized that the intervention group would exhibit higher abstinence rates. Secondary hypotheses for exploratory aims were that a) mCM would increase treatment engagement and retention, and that b) a longer-term, 3-month incentive would enhance the effectiveness of mCM.

## 2 Methods

### 2.1 Participants

All study procedures were approved by the Institutional Review Board at Durham VA Health Care System, including a waiver of HIPAA authorization for participant recruitment. Recruitment took place 2015 through 2017. Veterans experiencing homelessness face barriers to study participation (Bonevski et al., 2014), so multiple recruitment strategies were used. For mail-out recruitment, an electronic health record (EHR) data pull identified potentially eligible veterans, who received an introductory letter, which offered an option to opt out of any further study contact. Unless the patient opted out, a follow-up telephone call or in-person conversation was offered to determine preliminary eligibility. For clinician-led recruitment, clinicians in the VA Health Care for Homeless Veterans Program invited veterans to participate in the study. For exponential non-discriminative snowball sampling, ongoing study participants could refer up to 6 potential participants. When referred patients attended a baseline visit, the referring participant received $25.

Inclusion criteria included: homelessness, currently enrolled in the VA for healthcare, currently smoking ≥ 10 cigarettes daily, and willing to make a quit attempt in the next 30 days. “Homelessness” was defined as meeting any of the following criteria: 1) currently living in a shelter; 2) currently living in an institution that provides temporary residence; 3) currently living in a public/private place not designed for sleeping accommodation (e.g., car); 4) imminent loss of housing; or 5) a long-term period with housing instability (Perl, 2015).

Exclusion criteria included: current SUD not in remission, unmanaged psychotic symptoms, severely impaired hearing or speech (to ensure ability to complete telephone counseling), lack of interest in telehealth, or current pregnancy.

### 2.2 Procedure

Methodology for the current study was similar to a previously published pilot study (Carpenter et al., 2015). Potential participants attended an in-person screening session to complete informed consent, determine eligibility, and complete baseline measures. Then participants were randomized to two treatment groups (see below), as well as to a three-month abstinence incentive condition. Participants completed follow-up measures at 3-, 6-, and 12-months post-randomization, with self-report completed via telephone for participants who declined to attend in-person.

#### 2.2.1 mCM treatment group

Participants randomized to the treatment group received mCM, 5 weekly counseling sessions, and optional VA-prescribed tobacco cessation pharmacotherapy. The mCM intervention (Carpenter et al., 2015) was delivered using a study-owned smartphone (Android or iPhone) equipped with the mCM app, and a carbon monoxide (CO) monitor. Participants received an informational manual and training in the use of the smartphone, CO monitor, and mCM app. Participants practiced uploading two videos per day for one week prior to their self-appointed quit date. In the videos, they were asked to show themselves providing a breath sample with the CO monitor and showing the CO reading. They were compensated $1 for each video uploaded during this practice period.

Starting on a self-appointed quit date, participants were asked to upload videos verifying abstinence twice daily with at least 8 h between uploads. We used a previously published “escalating reinforcement schedule” for each subsequent video uploaded indicating abstinence (Carpenter et al., 2015). Compensation for confirmed abstinence ranged from $1 to $14.75, with the compensation resetting following a missed/non-abstinent reading. After 4 weeks of mCM, participants completed two weeks of CO monitoring without contingencies, with $1 compensation per video. The total possible amount earned for monitoring plus contingent payments was $528. In consideration of the range of CM in past studies (e.g., (Correia and Benson, 2006, Molina et al., 2022), we selected CM parameters to balance sufficient dose of the intervention with quick turnover study equipment to maximize participant recruitment.

Participants in the mCM group were also offered individual telehealth counseling (5 sessions of telehealth CBT for tobacco cessation; Carpenter et al., 2015) and optional pharmacotherapy. Pharmacotherapy included VA prescriptions for a 12-week course of nicotine replacement therapy (NRT; prioritizing nicotine patch plus as-needed oral NRT). If medically appropriate, participants could also receive a six-month prescription for bupropion 150 mg twice daily.

#### 2.2.2 Standard care treatment group

For participants randomized to the standard care treatment group, a consult was placed in the VA EHR for the local specialty smoking cessation clinic. This clinic bases its model on the QuitSmart™ Program (Shipley, 2009). The clinic program lasts 6 weeks and includes: 3 group counseling sessions led by a clinical psychologist, an individual telephone counseling session following their quit date, and pharmacotherapy prescribed by a psychiatrist (options for NRT, bupropion, or varenicline). Veterans were allowed to re-enroll in the program indefinitely. This control condition was selected for two reasons: 1) specialty care for tobacco cessation is recommended by clinical practice guidelines (Fiore et al., 2008), and 2) the local specialty smoking cessation clinic uses a model common across VA (Sherman et al., 2006).

#### 2.2.3 Three-month incentive

At baseline, all participants (regardless of treatment group) were randomized 1-to-1 to receive a $100 incentive for biochemically verified abstinence at the 3-month follow-up (confirmed by exhaled CO).

### 2.3 Measures

#### 2.3.1 Baseline measures

Participants completed demographic and tobacco-related measures, including average cigarettes per day and the Fagerström Test of Nicotine Dependence (Heatherton et al., 1991). Participants also completed the Structured Clinical Interview for DSM-5 Disorders (Michael et al., 2016) to determine number of psychiatric diagnoses (excluding nicotine dependence and counting all comorbid SUDs once).

#### 2.3.2 Prolonged smoking abstinence with lapses

The a priori primary outcome measure was biochemically verified prolonged smoking abstinence at 6 months post-randomization. Following recommendations for tobacco cessation outcomes (Piper et al., 2020), prolonged abstinence with lapses was defined as abstinence from smoking with the exception of lapses (i.e., some smoking not meeting the definition of a relapse). Smoking was allowable under this definition if it was a) <7 consecutive days and b) less than once a week for 2 consecutive weeks (not including a grace period of the first two weeks following the quit date) (McFall, et al., 2010, Hughes and Brandon, 2003). We additionally measured prolonged abstinence at 3- and 12-months post-randomization for longitudinal analysis. At each follow-up, the Timeline Follow-Back (TLFB) method (Lewis-Esquerre et al., 2005) was used to gather retrospective daily reports of smoking.

#### 2.3.3 Biochemical verification of abstinence

Biochemical verification of self-reported abstinence was required for all cessation outcomes, which is recommended to optimize scientific rigor and study validity (Benowitz et al., 2020). Saliva and CO samples were collected from participants who reported abstinence at each follow-up time point. Study staff attempted to collect salivary cotinine via mail for participants who declined in-person appointments and reported abstinence. While the original protocol specified cutoffs for CO (<6 ppm) and cotinine (<15 ng/mL) (Hughes and Brandon, 2003, McFall, et al., 2010) over the past decade, more stringent biochemical cutoffs to determine abstinence have been recommended for both CO and cotinine (Benowitz et al., 2020, Emery and Levine, 2016, Javors et al., 2011). A previously developed algorithm was adapted for the study (Benowitz et al., 2020, Cropsey et al., 2014, Javors et al., 2011). For participants who reported taking NRT or using vapes/e-cigarettes at follow-up, they were only marked as abstinent if they self-reported prolonged abstinence and provided a CO reading < 5 ppm. For participants who were not taking NRT/using vapes at follow-up, abstinence was based upon: a) self-reported abstinence, b) at least one sample indicating abstinence (CO < 5 ppm and/or cotinine < 6 ng/mL), and c) no biochemical samples indicating smoking (CO ≥ 5 ppm or cotinine ≥ 6 ng/mL).

#### 2.3.4 Seven-Day point prevalence abstinence

To assess seven-day point prevalence abstinence, participants were asked a single yes/no question, “Have you smoked a cigarette, even a puff, in the past 7 days?” Biochemical verification of point prevalence abstinence was identical to the process described above.

#### 2.3.5 Secondary outcomes

Intervention reach was defined as the proportion of participants who attended at least one treatment session. Similar to a past study, we defined video upload adherence for the mCM group as the proportion of possible videos that were uploaded during the treatment period (Carpenter et al., 2015). Costs for both groups were estimated in U.S. dollars ($) using standardized estimates. For the standard care group, clinicians were asked to provide time estimates for letters, scheduling, and appointments for a subset of 30 participants chosen randomly. For the mCM group, time estimates were assessed for time spent on: contacting, scheduling, training, counseling, and prescribing pharmacotherapy. The value of staff time was assessed using VA salary and benefits data. We also incorporated costs of materials (e.g., mCM web hosting, smartphone, contingency payments).

We calculated the incremental cost-effectiveness ratio (ICER; the additional intervention cost per additional life year saved) of the mCM intervention using estimates of intervention costs and effectiveness. The ICER is expressed as: $R=\frac{m_{\mathrm{CT}}-m_{\mathrm{CU}}}{m_{\mathrm{ET}}-m_{\mathrm{EU}}}$, where *m* denotes the estimated mean for *CT* (cost of mCM), *CU* (cost of standard care), *ET* (effectiveness of mCM), and *EU* (effectiveness of standard care). The effectiveness of each group was determined by calculating the average increase in life expectancy (quality-adjusted life years; QALY) due to tobacco cessation at the 6-month time point, accounting for: participant age and gender, 3% annual discount in quality of life per year, and 35% probability of relapse (Fiscella and Franks, 1996).

### 2.4 Analytic plan

Comparisons of all continuous variables were done with linear regressions. Comparisons of dichotomous variables were done with generalized linear models using a binomial distribution. Regarding the main prolonged abstinence time point (6 months) and other time points (3 and 12 months), odds ratios and 95% confidence intervals were computed from estimates obtained in generalized linear models. A secondary longitudinal analysis using generalized linear models was also conducted on prolonged abstinence across 3-, 6-, and 12-month time periods with predictors of treatment arm, time in months (standardized and centered), and the treatment arm-by-time interaction. Follow intent-to-treat analysis, participants with missing self-report abstinence or prolonged abstinence variables were designated as non-abstinent. All analyses were conducted in R version 4.0.4 (R Core Team, 2020).

## 3 Results

**Fig. 1:**
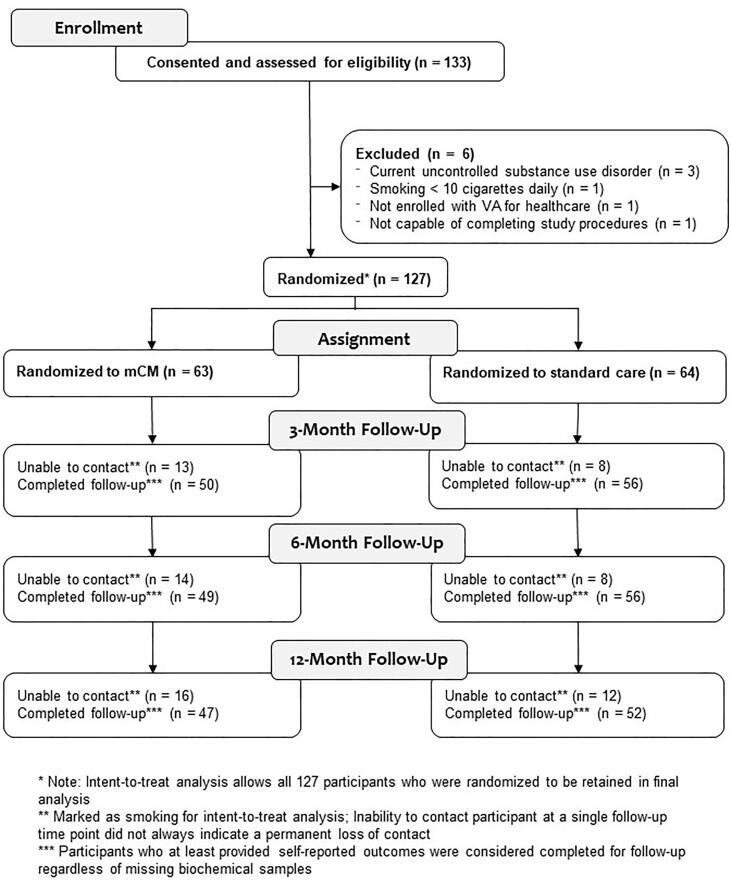
CONSORT Diagram, Smoking Cessation Randomized Trial among U.S. Military Veterans Experiencing Homelessness, Baseline through 12 Months Post-Randomization.

A total of 133 veterans attended an in-person screening session to assess study eligibility. Of those, 127 veterans were randomized, 63 to mCM and 64 to standard care (see Fig. 1; CONSORT Diagram). See Table 1 for sample characteristics.

**Table 1:** Baseline participant characteristics, smoking cessation trial of military veterans experiencing homelessness in the Southeastern U.S.

Variable	Standard Care Treatment *N* = 64 *M (SD)* *n* (%)	mCM Treatment Condition *N* = 63 *M (SD)* *n* (%)	Univariate Difference Test
Age	55.72 (7.49)	53.76 (10.26)	*t* = 1.23, *p* =.22
Gender			Fisher’s Exact *p* =.49
Men	61 (95.3%)	57 (90.5%)	
Women	3 (4.6%)	6 (9.5%)	
Race			Fisher’s Exact *p* =.35
American Indian/Alaska Native	2 (3.1%)	0 (0%)	
Black	44 (68.8%)	39 (61.9%)	
Multiracial	7 (10.9%)	7 (11.1%)	
White	11 (17.2%)	17 (27.0%)	
Hispanic/Latino Ethnicity	2 (3.1%)	2 (3.2%)	Fisher’s Exact *p* =.99
Service-Connected Disability	27 (42.2%)	22 (34.9%)	*χ^2^* = 0.44, *p* =.51
Housing Status			*χ^2^* = 1.55, *p* =.82
Shelter	8 (12.5%)	8 (12.7%)	
Street/Woods/Car	3 (4.7%)	3 (4.8%)	
Transitional Housing/ Substance Use Treatment Facility/Group Home/ Assisted Living	18 (28.1%)	23 (36.5%)	
Temporarily Staying with Family/Friends	22 (34.4%)	16 (25.4%)	
Other Current or Recent Housing Instability	13 (20.3%)	13 (20.6%)	
Years of Education	13.12 (1.96)	13.67 (2.31)	*t* = 1.43, *p* =.16
Nicotine Dependence^a^	8.08 (1.45)	8.10 (1.33)	*t* = 0.07, *p* =.94
Cigarettes per Day	15.35 (7.30)	16.65 (8.47)	*t* = 0.92, *p* =.36
Number of *DSM-5* Diagnoses	1.19 (1.02)	1.33 (1.23)	*t* = 0.73, *p* =.47

^a^ Fagerstrom Test of Nicotine Dependence.

Note. Fisher’s Exact Test used in place of Chi-Square test-of-independence when cell sizes are small.

### 3.1 Main Outcome: Prolonged smoking abstinence with lapses

**Fig. 2:**
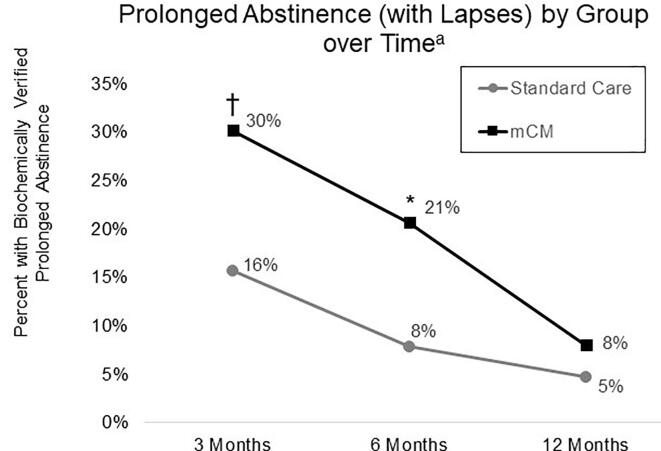
Proportion of U.S. Military Veteran Participants with Biochemically Verified Prolonged Smoking Abstinence (with Lapses) by Treatment Group at 3, 6, and 12 Months Post-Randomization. † *p* =.05, * *p* <.05 ^a^ Across time points, the mCM group had significantly higher biochemically verified smoking abstinence, *OR* = 2.34 (95% *C.I.* 1.18–4.61), *p* <.01].

As shown in Fig. 2, participants in the mCM group were significantly more likely to demonstrate prolonged abstinence at 6-month follow-up (*OR* = 3.07, 95% *CI* [1.02, 9.20], *p* =.04). At 3-month follow-up, the mCM group was marginally more likely to meet criteria for prolonged abstinence at the 3-month follow-up (*OR* = 2.33, 95% *CI* [0.98, 5.53], *p* =.05), and at the 12-month follow-up, no group difference in prolonged abstinence was detected (*OR* = 1.75, 95% *CI* [0.40, 7.67], *p* =.46).

In the longitudinal model, there was a significant effect for treatment group (*OR* = 2.34, 95% *CI* [1.18–4.61], *p* <.01), with the mCM group being more likely to demonstrate prolonged abstinence (Fig. 2).

### 3.2 Point prevalence smoking abstinence

**Fig. 3:**
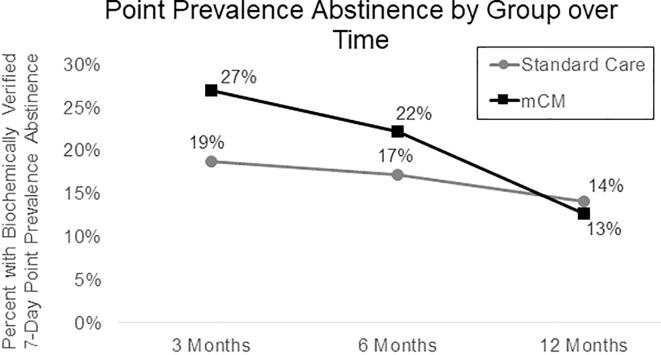
Proportion of U.S. Military Veteran Participants with Biochemically Verified Seven-Day Point Prevalence Smoking Abstinence by Treatment Group at 3, 6, and 12 Months Post-Randomization. ^a^ Across time points, there was no significant difference in seven-day point prevalence abstinence between groups (*OR* = 1.25, 95% *CI* [0.74–2.13], *p* =.40).

Across time points, there was no significant difference in seven-day point prevalence abstinence between groups (*OR* = 1.25, 95% *CI* [0.74–2.13], *p* =.40). As shown in Fig. 3, contrasts at each time point revealed no significant differences at any time point: 3-month follow-up, *OR* = 1.60, 95% *CI* [0.69, 3.70], *p* =.27); 6-month follow-up, *OR* = 1.38, 95% *CI* [0.57, 3.32], *p* =.48); 12-month follow-up, *OR* = 0.89, 95% *CI* [0.32, 2.47], *p* =.82).

### 3.3 Three-Month abstinence incentive

Randomization to the 3-month abstinence incentive condition at baseline was not related to abstinence at 3-months (*OR* = 0.62, 95% *C.I.* [0.14, 2.78], *p* =.53). There was no detectable longitudinal interaction effect between incentive randomization group and treatment group.

### 3.4 Effectiveness and Cost-Effectiveness

Based on 6-month outcomes, the number needed to treat (NNT) was 7.7 for the mCM versus standard care groups. In other words, 8 veterans would need to receive the mCM treatment rather than the standard care for one additional veteran to achieve tobacco abstinence at 6 months. Regarding increase in life years, the standard care group saved on average 0.17 QALY (or 0.10 QALY if assuming a 3% discount in quality of life per year). The mCM group on average saved 0.52 QALY (or 0.28 QALY if assuming a 3% discount in quality of life per year). The estimated mean treatment cost per patient was $226 for the standard care group and $618 for the mCM group. In terms of incremental cost-effectiveness, the mCM intervention was estimated to cost an additional $1,133 per QALY saved (or $2,217 per QALY if assuming a 3% annual discount in quality of life per year) over and above standard care.

### 3.5 Secondary outcomes

#### 3.5.1 Intervention reach

Results indicated that participants in the mCM group were more likely to complete at least one treatment session (95%, *n* = 60) compared to the standard care group (58%, *n* = 37; *χ^2^*(1) = 22.62, *p* <.001). At 3-month follow-up, groups did not differ in their self-report of taking either NRT (mCM: n = 32, 50.8%; standard care: n = 35, 54.7%) or oral medication (mCM: n = 13, 20.6%; standard care: n = 13, 20.3%).

#### 3.5.2 mCM video upload adherence and earnings

The mean amount of abstinence incentives earned in the mCM group via video uploads was $153.06 (Range: $0-$528.00, SD = $180.02). Within the mCM group, 52 (82.5%) participants uploaded at least one video aside from the practice video completed in-session. Among those with at least one self-uploaded video, mean video upload adherence rate was 66.01 percentage points (*SD* = 32.77). mCM video upload adherence significantly predicted prolonged abstinence at 6-month follow-up (*OR* = 1.06, 95% CI [1.01, 1.11], *p* =.04).

### 3.6 Post-Hoc exploratory analysis findings

Two post-hoc exploratory analyses were completed due to observations reported by study staff. In analyzing abstinence, a high rate of discordance between self-report and biochemical verification was observed and explored post-hoc (Table 2). Notably, at 3- and 6-month follow-up, those in the mCM group were significantly more likely to provide biochemical samples that supported their self-report of abstinence. Discordance in biochemical verification of smoking was largely driven by samples indicating smoking rather than missing samples.

**Table 2:** Biochemical Verification of Abstinence among U.S. Military Veterans Self-Reporting Prolonged Abstinence at 3, 6, and 12 Months Post-Randomization by Treatment Group.

	Bioverified Abstinent	Not Bioverified Abstinent*	Chi-Square Difference Test
**3-Month Follow-Up**			
Treatment Group (n = 27)	19 (70.37%)	8 (29.63%)	*χ*^2^ = 6.03, *p* =.01
Control Group (n = 27)	10 (37.04%)	17 (62.96%)	
Total Self-Reporting Abstinence (n = 54)	29 (53.70%)	25 (46.30%)	
**6-Month Follow-Up**			
Treatment Group (n = 28)	13 (46.43%)	15 (53.57%)	*χ*^2^ = 4.49, *p* =.03
Control Group (n = 26)	5 (19.23%)	21 (80.77%)	
Total Self-Reporting Abstinence (n = 54)	18 (33.33%)	36 (66.66%)	
**12-Month Follow-Up**			
Treatment Group (n = 25)	5 (20.00%)	20 (80.00%)	*χ*^2^ = 1.10, *p* =.29
Control Group (n = 30)	3 (10.00%)	27 (90.00%)	
Total Self-Reporting Abstinence (n = 55)	8 (14.55%)	47 (85.45%)	

* Number of participants by group who reported prolonged abstinence, but whose biochemical verification indicated smoking.

Study staff also observed long wait times for clinic appointments in the standard care condition. Thus, a post hoc exploratory analysis of days until first treatment session was analyzed. Groups differed in the number of days from randomization to first treatment session, mCM group: 13.95 days (*SD* = 20.36); standard care group: 49.24 days (*SD* = 46.84); *t*(1 2 5) = 7.77 (*p* <.01).

## 4 Discussion

This study examined the clinical effectiveness of an intensive multi-modal smoking cessation intervention including mCM, CBT, pharmacotherapy, and long-term abstinence incentives compared to standard care among veterans who smoke and are experiencing homelessness. Over time, prolonged abstinence rates were higher in the mCM treatment group compared to standard care. Additionally, considering common benchmarks, the cost of mCM at 6 months compared to standard care was modest given its level of effectiveness (Cohen and Reynolds, 2008, Owens, 1998).

Study findings support the short-term cost-effectiveness of mCM and are consistent with previous evidence of treatment effects waning over time (Baggett et al., 2018a, Baggett et al., 2018b, Rash et al., 2018). Compared to other non-CM cessation trials among homeless populations, the 6-month quit rate was high (Vijayaraghavan et al., 2020). In the current study, prolonged abstinence was initially high in the mCM group (30% at 3 months post-randomization) but declined by the 12-month follow-up (8%). Although a similar pattern of results was observed in the 7-day point prevalence outcome, this finding was not significant, possibly due to high rates of 7-day abstinence without longer prolonged abstinence in the standard care group.

For tobacco users experiencing homelessness, unique barriers may have influenced diminishing effects of CM over time. In this population, subsistence difficulties (e.g., finding shelter, food, bathrooms) are linked with lower odds of smoking abstinence (Baggett et al., 2018a, Baggett et al., 2018b). Qualitative research has echoed these findings, indicating that homelessness-related stressors represent a barrier to smoking cessation (Pratt et al., 2019). In healthcare settings, individuals experiencing homelessness report being stigmatized, treated as invisible, and treated with disrespect (Martins, 2008). Moreover, systemic racism is often inextricably linked to homelessness-related discrimination and stigma (Paul et al., 2020, Weisz and Quinn, 2018). It will be important for future studies to explore ways to address these social determinants of tobacco use in order to sustain abstinence rates achieved with mCM.

In exploratory post-hoc analyses, participants in the mCM group were more likely to complete a first treatment session, suggesting that mCM may have enhanced treatment engagement. However, group differences in counseling format (i.e., 3 group sessions versus 5 individual sessions) may have influenced engagement. Exploratory findings also indicated discrepancies in self-reported smoking abstinence compared to biochemically verified abstinence, which has been found in prior studies (Patrick et al., 1994). If biochemical verification had not been used, no group difference in treatment effect would have been detected. This group difference in biochemical verification discrepancy was likely due to the mCM group’s repeated exposure to verification of abstinence during mCM monitoring.

Interestingly, long-term incentives at 3-month follow-up were not associated with prolonged abstinence at any follow-up time point. It is unclear what led to the lack of effect in this long-term incentive, since there is no detectable association between effectiveness of financial incentives and the amount of the incentive offered (Notley et al., 2019).

This study has some limitations. First, it is possible that structural inequities in exposure to secondhand smoke and/or CO from air pollution incorrectly lowered rates of biochemically verified abstinence in our sample (Houston et al., 2004, Rowangould, 2013). Second, an unintended consequence of using standard care as a control condition was that treatment was delayed in this group. However, if anything, this delay likely strengthened the measured effect of the control condition due to a shorter time from treatment to follow-ups. Regarding snowball sampling, data on the number of referrals yielded per seed were not collected for analysis. Additionally, substance use was not monitored across the study to ensure that reducing smoking did not increase substance use among participants. However, prior evidence suggests that tobacco abstinence is linked with lower odds of heavy drinking and no effect on drug use (Reitzel et al., 2014). And finally, there was a low proportion of women in the study, which limits generalizability across genders. Although only 9% of unhoused veterans are women (Henry et al., 2017), it is crucial to know the effectiveness of cessation interventions with this population given their unique experiences.

## 5 Conclusion

This study provides evidence that CM has short-term effectiveness for smoking abstinence among veterans experiencing homelessness. However, abstinence rates declined from 6 to 12 months, with no discernable group difference at 12-month follow-up. It is crucial to continue to identify ways to sustain tobacco abstinence rates yielded by CM interventions. Future tobacco cessation studies among people experiencing homelessness should test whether abstinence outcomes may be improved by addressing social determinants of tobacco use and structural discrimination. If this approach were combined with a powerful short-term intervention such as mCM, this could help to maintain abstinence long-term.

## CRediT authorship contribution statement

**Sarah M. Wilson:** Investigation, Resources, Writing – original draft, Visualization, Writing – review & editing. **Dan V. Blalock:** Formal analysis. **Jonathan R. Young:** Writing – original draft, Writing – review & editing. **Sarah C. Griffin:** Visualization, Writing – review & editing. **Jeffrey S. Hertzberg:** Software, Methodology, Data curation. **Patrick S. Calhoun:** Conceptualization, Funding acquisition, Methodology, Supervision, Investigation, Writing – review & editing. **Jean C. Beckham:** Conceptualization, Methodology, Funding acquisition, Supervision, Investigation, Project administration.

## Declaration of Competing Interest

The authors declare that they have no known competing financial interests or personal relationships that could have appeared to influence the work reported in this paper.

## Data Availability

Data will be made available on request.
